# Mutational analysis of oncogenic *AKT* E17K mutation in common solid cancers and acute leukaemias

**DOI:** 10.1038/sj.bjc.6604212

**Published:** 2008-04-08

**Authors:** M S Kim, E G Jeong, N J Yoo, S H Lee

**Affiliations:** 1 Department of Pathology, College of Medicine, The Catholic University of Korea, Seoul, Korea

**Keywords:** AKT1 E17K, mutation, oncogene

## Abstract

Mounting evidence indicates that alterations of AKT signalling play important roles in cancer development. An earlier study discovered an oncogenic *AKT1* gene mutation (*AKT1* E17K) in breast, colorectal and ovarian cancers. The aim of this study was to see whether the *AKT1* E17K mutation is common in breast, colorectal, lung, gastric and hepatocellular carcinomas and acute leukaemias. We analysed the presence of the *AKT1* E17K mutation in 731 cancer tissues by a single-strand conformation polymorphism assay. In addition, we analysed the corresponding sequences of *AKT1* E17K in *AKT2* and *AKT3* genes. Overall, we detected the four *AKT1* E17K mutations in the breast cancers (4/93; 4.3%), but none in other cancers. There was no *AKT2* or *AKT3* mutation in the cancers. This study demonstrated that the *AKT1* E17K mutation occurs in breast cancers at a low frequency, and that it is rare in other common cancers, including colorectal, lung, gastric and hepatocellular carcinomas and acute leukaemias. Despite the confirmed oncogenic function of the *AKT1* E17K, the rare incidences of the mutation suggest that it may not play a crucial role in the development of the most common types of human cancers.

Protein kinases regulate cell signalling pathways mediating a number of processes in cell proliferation, differentiation and survival (Gschwind *et al*, 2004). AKT1 (also known as protein kinase B) is a subfamily of serine/threonine protein kinases, and the *AKT* genes are the mammalian equivalent of murine viral oncogene v-akt (Testa and Bellacosa, 2001). There are three isoforms of the AKT (AKT1, AKT2 and AKT3), and each AKT member contains an N-terminal pleckstrin homology (PH) domain, a short *α*-helical linker and a C-terminal kinase domain (Testa and Bellacosa, 2001). AKTs are major downstream targets of growth factor receptors that signal through phosphatidylinositol 3-kinase (Testa and Bellacosa, 2001). Mounting evidence exists that activation of AKT proteins is important in cancer development (Muise-Helmericks *et al*, 1998; Dimmeler *et al*, 1999; Khwaja, 1999; Ozes *et al*, 1999; Mende *et al*, 2001; Wei *et al*, 2001). PTEN inhibits AKT activation and works as a tumour suppressor (Testa and Bellacosa, 2001). Inactivating mutation of *PTEN* and activating mutation of *PIK3CA* have been detected in many human cancers and have been known as major activating mechanisms of AKT-signalling pathway in cancers (Testa and Bellacosa, 2001; Samuels *et al*, 2004). By contrast, activating mutation of *AKT* genes has not been widely reported in human cancers.

Recently, a research group discovered a recurrent somatic mutation of *AKT1* gene in human breast, colorectal and ovarian cancers (Carpten *et al*, 2007). The *AKT1* mutation was a missense mutation that substituted an amino acid (E17K) in the PH domain of AKT1. The *AKT1* E17K mutation was found in 5 out of 61 (8.2%) breast cancers, 3 out of 51 (5.9%) colorectal cancers and 1 out of 50 (2.0%) ovarian cancers (Carpten *et al*, 2007). The E17K mutation was mutually exclusive with respect to *PIK3CA* mutation and loss of PTEN expression (Carpten *et al*, 2007). Functionally, the *AKT1* E17K mutation stimulates AKT signalling, induces cellular transformation and produces leukaemia in mice, strongly suggesting that this mutation may play a crucial role in cancer development (Carpten *et al*, 2007).

To further characterise the *AKT1* E17K mutation in human cancers, the following questions were investigated in this study: (a) whether human cancer tissues from various histologic origins have the *AKT1* E17K mutation; (b) whether there is any ethnic difference of the *AKT1* E17K mutation incidence in breast and colorectal cancers; and (c) whether any corresponding mutation of *AKT1* E17K occurs in *AKT2* or *AKT3* genes.

## MATERIALS AND METHODS

We analysed *AKT1* for the detection of the *AKT1* E17K mutation in methacarn-fixed tissues of 93 breast ductal carcinomas, 104 colorectal adenocarcinomas, 180 gastric adenocarcinomas, 68 hepatocellular carcinomas and 157 non-small cell lung cancers (NSCLCs), and fresh non-fixed fresh tissues of 129 adulthood acute leukaemias by polymerase chain reaction (PCR)-single strand conformation polymorphism (SSCP) analysis. All of the cancer patients were Korean. Approval for this study was obtained from the Institutional Review Board of the Catholic University of Korea, College of Medicine. The gastric carcinomas consisted of 40 early and 140 advanced gastric carcinomas. The breast carcinomas consisted of 15 ductal carcinomas *in situ* and 78 invasive ductal carcinomas. The NSCLCs consisted of 74 adenocarcinomas, 70 squamous cell carcinomas, 3 adenosquamous carcinomas and 10 large-cell carcinomas. The acute leukaemias consisted of 95 acute myelogenous leukaemia, 33 acute lymphoblastic leukaemia (ALL) (29 B-ALL and 4 T-ALL) and 1 undifferentiated acute leukaemia. For the solid tumours, malignant cells and normal cells were selectively procured from haematoxylin and eosin-stained slides by a microdissection as described previously (Lee *et al*, 1999), whereas for the leukaemia non-fixed fresh bone marrows were directly used. Because the *AKT1* E17K mutation was detected in the exon 3, genomic DNA each from tumour cells and corresponding normal cells were amplified with one primer pair covering the exon 3. Radioisotope ([^32^P]dCTP) was incorporated into the PCR products for detection by SSCP autoradiogram. After SSCP, bands showing mobility shifts were re-amplified and sequenced by a capillary automatic sequencer.

We also analysed *AKT2* exon 3 and *AKT3* exon 2 by the same PCR-SSCP method. In this approach, we used 45 breast ductal carcinomas, 45 colorectal adenocarcinomas, 45 gastric adenocarcinomas, 45 hepatocellular carcinomas, and 45 NSCLCs and 45 adulthood acute leukaemias.

## RESULTS AND DISCUSSION

**Figure 1:**
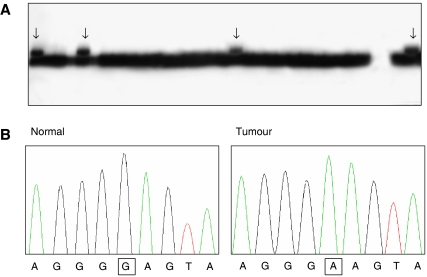
Representative data on *AKT1* E17K mutation in the cancers. (**A**) The PCR products of the exon 3 from breast cancers were visualised on SSCP. The arrows indicate aberrant bands as compared with the wild-type bands. (**B**) One of the aberrant bands on the SSCP was sequenced. The boxes indicate nucleotide changes in the tumour DNA as compared with normal tissue DNA.

The PCR-SSCP analysis of the *AKT1* exon 3 in the 731 cancers identified aberrantly migrating bands in four breast cancers (4/93; 4.3%), but not in other cancers (Figure 1A). All of the four breast cancers were invasive ductal carcinomas. DNA sequence analysis revealed that the aberrant bands represented an identical mutation (c.49G>A (p.E17K)) (Figure 1B). None of the normal samples from the patients with the *AKT1* E17K mutation showed evidence of mutation by the SSCP, indicating that the mutations had risen somatically. We also confirm the mutation by a direct DNA sequencing in the four breast cancers (data not shown). In addition, we performed direct DNA sequencing analyses for the *AKT1* E17K mutation in 120 cases of the cancers and found no additional *AKT1* E17K mutation in the cancers (data not shown).

Next, we attempted to find out the corresponding mutation of *AKT1* E17K in both *AKT2* and *AKT3* genes. However, the SSCP from the tumours did not reveal any aberrantly migrating band compared with the wild-type bands from the normal tissues (data not shown). We repeated the experiments twice, including tissue microdissection, PCR, SSCP and DNA sequencing analysis to ensure the specificity of the results and found that the data were consistent.

Because the previous study showed modest incidences of *AKT1* E17K mutation (up to 8.2%) in a wide range of the cancers (Carpten *et al*, 2007), we expected to detect comparable *AKT1* E17K mutations in our cancer specimens. However, we detected the *AKT1* E17K mutation in the breast cancers (4.3%), but not in other cancers. Statistically, there is no difference of the *AKT1* E17K mutation frequency in the breast cancers between the previous study and our study (Fisher's exact test, *P*=0.253). By contrast, there is a significant difference of the *AKT1* E17K mutation frequency in the colorectal cancers between the previous study and our study (Fisher's exact test, *P*=0.034), suggesting a possibility that a racial difference might exist. However, because the previous reports did not describe the ethnicity of the patients (Carpten *et al*, 2007), it is not possible to conclude whether the difference arose from a racial difference. Together, these data suggest that the *AKT1* E17K mutation may be rare in common human cancers besides breast cancers. Also we found neither *AKT2* nor *AKT3* mutation in the corresponding sequences to the *AKT1* E17K, indicating that mutational alteration of neither *AKT2* nor *AKT3* contributes to cancer development.

Because the most impressive examples of recent cancer therapies target activated kinases by genetic alterations, cancer researches have focused on evaluating kinases as promising molecular targets for cancer treatment (Druker *et al*, 2001; Fukuoka *et al*, 2003; Gschwind *et al*, 2004; Paez *et al*, 2004). The discovery of the *AKT1* E17K mutation in cancers raised a possibility to treat cancers by targeting the mutated AKT1. However, our data confirm the earlier data only in breast cancers, but not in other types of common cancers. Therefore, the present study suggested that the *AKT1* E17K mutation should further be analysed in a wider range of cancers.
